# Cardiac lymphoid hyperplasia is the same as cardiac nodularity‐like appearance

**DOI:** 10.1002/deo2.95

**Published:** 2022-03-24

**Authors:** Toshihiro Nishizawa, Shuntaro Yoshida, Osamu Toyoshima

**Affiliations:** ^1^ Department of Gastroenterology Toyoshima Endoscopy Clinic Tokyo Japan; ^2^ Department of Gastroenterology and Hepatology Narita Hospital, International University of Health and Welfare Chiba Japan

Dear Editor,

We have read the article “endoscopic findings of cardiac lymphoid hyperplasia and *Helicobacter pylori* infection status” by Adachi and his colleagues with great interest.^1^ The photos of cardiac lymphoid hyperplasia were beautiful, and we easily noticed that cardiac lymphoid hyperplasia was the same as cardiac nodularity‐like appearance which we recently reported.^2^, ^3^ In fact, we also have reported a similar phenomenon. We have found that this endoscopic finding was associated with *H. pylori* infection^2^ and was improved by *H. pylori* eradication.^3^ Nodularity mainly appears in the antral mucosa with *H. pylori* infection, and antral nodularity is also improved by *H. pylori* eradication.^4^ The nodularity as the endoscopic term is included in the Kyoto classification, and the pathophysiology is lymphoid hyperplasia.^5^ So, the term may be better to be used to correspond to this. We considered that cardiac nodularity‐like appearance should be used as an endoscopic finding, and the pathophysiology is cardiac lymphoid hyperplasia. Either way, the term should be unified.

## CONFLICT OF INTEREST

The authors declare no conflict of interest.

## FUNDING INFORMATION

None
